# The House Fly as Distributor of Disease

**Published:** 1887-08

**Authors:** M. Moore

**Affiliations:** Scranton, Miss.


### THE HOUSE-FLY AS A DISTRIBUTOR OF DISEASE.

BY M. MOORE, M. D., OF SCRANTON, MISS.

I have for several years, as a practitioner in both cities and rural districts, looked after the cause of endemic and epidemic diseases. The endemic is hard to define. I believe they are natural, and will at all times appear when local causes are sufficient to produce them. I believe that the common house-fly is the great distributor of every epidemic that is or has been introduced to our country; and, by close investigation, it will be found to be a distributor of small-pox, measles, scarlet fever, yellow fever, and many infectious diseases, so-called. I call for a microscopical examination of this inscrutable fly.

